# Implementing new models of care: Lessons from the new care models programme in England

**DOI:** 10.1177/2053434518770613

**Published:** 2018-04-16

**Authors:** Anna Starling, on behalf of The Health Foundation

**Affiliations:** The Health Foundation, London, UK

**Keywords:** Health services research, integrated care, primary health care, care pathways, quality of care, health care reform

## Abstract

In 2014, the body that leads the National Health Service in England published a new strategic vision for the National Health Service. A major part of this strategy was a three-year-long national programme to develop new care models to coordinate care across primary care, community services and hospitals that could be replicated across the country. Local ‘vanguard sites’ were selected to develop five types of new care model with support from a national team. The new care models programme provided support for local leaders to enable them to collaborate to improve care for their local populations. We interviewed leaders in the vanguard sites to better understand how they made changes to care locally. Drawing on the insights from these interviews and the literature on cross-organisational change and improvement we devised a framework of 10 lessons for health and care leaders seeking to develop and implement new models of care. The framework emphasises the importance of developing relationships and building capability locally to enable areas to continuously develop and test new ideas.

## Introduction

For the National Health Service (NHS) in England, ambitions to better integrate around the needs of those who need health and social care are not new. A 1972 white paper acknowledged that ‘a single family, or an individual, may… need many types of health and social care and those needs should be met in a coordinated manner’.^1^ In the last 46 years, some areas of England have made progress towards coordinated care but these changes have not happened systematically. This is despite successive ‘top-down’ national approaches to try and make this happen through legislation, targets and funded pilot programmes.

In 2014, the body that leads the NHS in England^2^ published a new strategic vision for the NHS.^3^ A major part of this strategy was a three-year-long national programme to develop ‘new care models’ to coordinate care across primary care, community services and hospitals that could be replicated across the country. These models were intended to contribute towards achieving the triple aim of improved patient care, reduced cost and better population health.^4^

In a departure from the long history of ‘top-down’ national initiatives with similar aims, the new care models programme aimed to reconcile ‘top-down’ and ‘bottom-up’ approaches to achieving change. Senior leaders of the programme wanted to be more involved than providing funding for and evaluations of pilots, but were mindful of the risks of crowding out local initiative, innovation and ownership. To do this, 50 local ‘vanguard sites’ were selected to develop a range of different types of new care model with support from the national programme led by NHS England. As the programme entered its final year in 2017, we undertook a qualitative piece of research to better understand how the vanguard sites approached the implementation of new models of care and the implications for the future.^5^

## The programme

**Figure 1.**
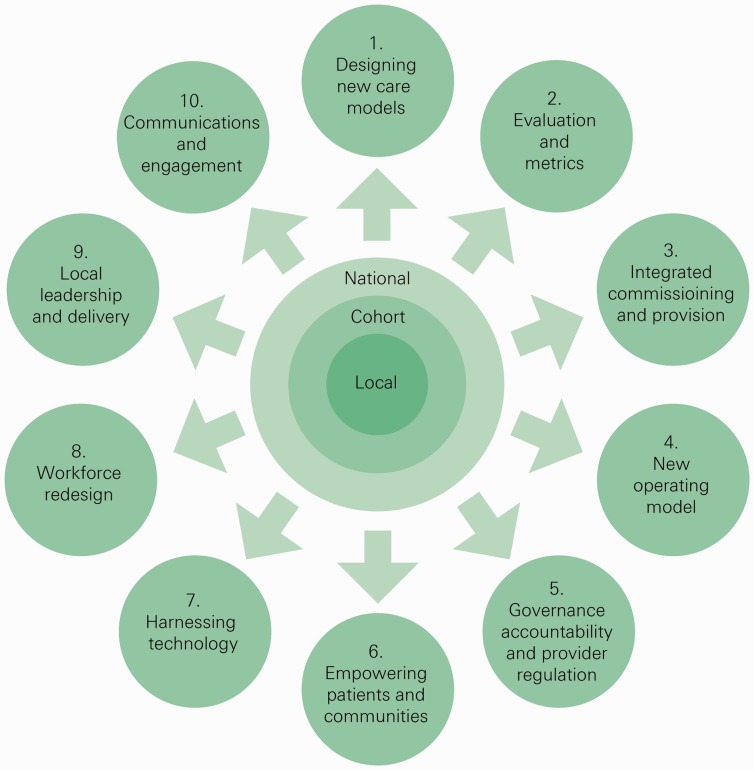
The enablers from the national programme team.

National leaders of the new care models programme outlined five categories of new care model (to help create blueprints for other areas to learn from) but otherwise sought to limit prescribing the ‘what’ of change.^6^ Vanguard sites received modest funding, ranging from £500,000 to £8m per site per year.^7,8^ In addition, the national programme also aimed to offer what Allcock et al.^9^ have described as proactive support – support that focuses on enabling local systems to make the changes needed and gives them licence to test and refine new ideas. That national support package of help and guidance covered 10 areas (see Figure 1) which were co-produced with sites.

The national programme placed considerable emphasis on designing evaluation for vanguard sites to help identify what worked and how and for this to be shared across the country.^10^ Sites were given funding to procure local quantitative and qualitative evaluations as well as access to central evaluation support. This included specialist analysis from the Improvement Analytics Unit (IAU) – a relatively new partnership between NHS England and independent charity the Health Foundation that aims to provide rapid-cycle quantitative evaluation to show whether local change initiatives are improving care and efficiency.^11^

## The models and early results

At its inception in January 2015, the new care models programme invited expressions of interest for three population-based categories of new care model: enhanced health in care homes, multi-specialty community providers and primary and acute care systems (Table 1). Of the 50 vanguard sites 29 were selected to be one of these three models, based on being able to evidence previous progress of making changes across their health and care system.^12^ The three models aimed to improve care for populations whose care would benefit from greater coordination of services: predominately older people, those with chronic conditions and those identified as being high risk of admission to acute care. The vanguard sites therefore sought to reflect the needs of these target populations by redesigning services with a focus on the provision of care outside of hospitals.

**Table 1.** Profiles of the three vanguard model types.

Model	Description	Number of sites	Initial population	Common services	Population size
Enhanced health in care homes	A model that focuses on connecting care homes into health care	6	Care home residents, Older people who are in community beds or recipients of care in the community	Enhanced primary care for care homes,Multidisciplinary teams,Reablement and rehabilitation,Improved end of life and dementia care,Improved transfers	2500–200,000
Multispecialty community providers	An integrated provider of out-of-hospital care	14	People with long-term conditions, Older people,Other vulnerable groups in the population identified at high risk of admission to hospital	Integrated community teams,Enhanced primary care services,Specialist care in the community/at home,Rapid response teams,Self-care and prevention services	100,000-300,000 (organised into localities of 30,000-50,000)
Primary and acute care systems	A model that integrates the provision of hospital, primary, community and mental health services	9	People with long-term conditions,Older people,Other vulnerable groups in the population identified at high risk of admission to hospital,Urgent and emergency care patients,Patients with elective care needs	Integrated community teams,Specialist care in the community/at home,Redesigned urgent care,Rapid response teams,Enhanced primary care services,Self-care and prevention services	250,000-300,000 (some organised into localities of 30,000-50,000)

NHS England have reported that the vanguard sites have seen lower growth in emergency hospital admissions and emergency inpatient bed days than the rest of England.^13^ The IAU evaluations have thus far published promising findings in one area in the East Midlands,^14^ although changes to care in another vanguard site in the North East did not appear to have the intended impact.^15^ Further evaluations are planned for publication in 2018 as well as early results from the full national evaluation of the programme.^16^

## Building local capability for integrated care

To understand how vanguard sites made changes locally and make some of this learning available for others in the short term, we explored what the site leaders thought, felt and did,^17^ interviewing 45 middle-to-senior clinical and non-clinical leaders and evaluators across eight of the vanguard sites.

We found that these leaders used informal partnerships to develop collaborative relationships and redesign care. Often building on years of work before the programme began, the sites tested many different cross-organisational pathways simultaneously. This involved creating new teams and roles, innovative ways of sharing information and new locations for providing treatment.

These activities were coordinated through local overarching programmes. By bringing together the different strands of work in this way, teams aimed to avoid the pitfalls that can occur when interventions are designed in isolation which Salisbury et al.^18^ describe as potentially leading to duplications, inefficiency and confusion for patients. The sites considered formal changes to governance and organisational forms, but not until later in the development of the new care model. This was another important departure from many previous approaches to change in the English NHS, which have typically started with changes to organisational structures, legislation or contracts.

**Figure 2.**
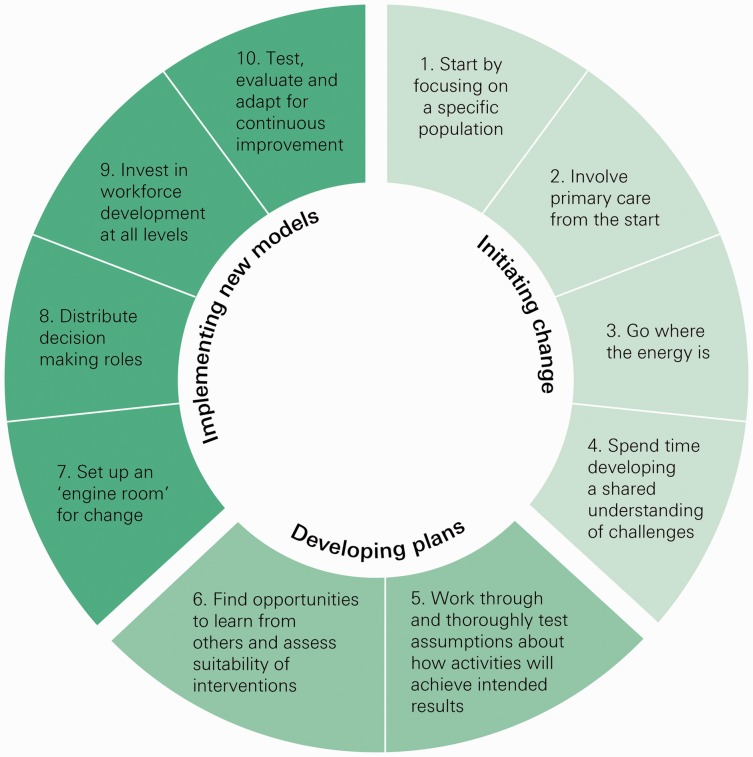
Ten lessons to support new care models locally.

We drew on common themes from the interviews and the literature on cross-organisational change and improvement to devise a framework of 10 lessons (Figure 2) that might be helpful for others seeking to develop and implement new models of care. They are categorised into three stages: initiating change, developing plans and implementing new models. These stages and their lessons are interconnected and should not be understood as strictly linear. The complexity of local health and care systems means there will be inherent messiness and unpredictability.^19^

Lessons 1 to 6 suggest first focusing on a specific population and its needs, building in time for developing a shared understanding of problems^20^ and co-designing new pathways with patients and their families. Gathering data and bringing stakeholders together is also a key to ensuring that areas can understand and adapt to local context. This national programme mandated that the sites develop logic models^21^ as part of this. Our interviewees described this as helpful ensuring they challenged assumptions and drew out risks and enabling factors before rushing to implementation.

Change cannot happen without the right capabilities and skills. Lesson 7 describes the benefit of bringing some of this expertise together locally into programme teams that include project management, quality improvement, data analysis, communication and administrative expertise. In the literature on implementation, these central teams are a key factor in achieving change when embarking on unfamiliar activities.^22,23^ Many of the vanguard sites used the additional funding from the national programme to backfill staff vacancies to enable those already working in the local health and care system to be part of these teams. We heard that this was important as it helped to create confidence among stakeholders and increase how quickly teams could start, thanks to their existing knowledge of the area.

Lessons 8–10 emphasise that evaluation to understand what works and why must also be a core component of plans and should be considered from the outset. This requires areas to build capability locally both for the collection and analysis of data. Staff working in the services where these changes are taking place may need assistance to enable them to reflect on when lessons have not worked and to change track where necessary which also requires distributing decision making roles and investment in workforce development at all levels. In the vanguard sites, we heard how they could do this building on the initial work of developing logic models and learning from the local evaluators whose presence was a programme requirement. The 10 lessons are set out in full in the learning report of our research.

## Value for money and bigger systems

The ‘vanguard sites’ participation in the new care models programme gave them the time and space to approach change in this way as opposed to being required to deliver results immediately. However, we heard that this space became less readily available in the final year of the programme as expectations from the national programme became more focused on demonstrating reductions in emergency admissions and value for money. This was also seen in Ererns et al.’s evaluation of a previous national initiative in England, the integrated care pioneers.^24^

The new care models are now meant to spread rapidly to new areas with a target for achieving 50% coverage across England by December 2020.^25^ This will be challenging in what is an increasingly financially squeezed health and social care context^26^ and with far less central support available for new areas seeking to make these changes than was given to the vanguard sites.

In 10 areas of England, larger geographical areas that have shown progress in implementing new care models have been designated as integrated care systems – a status that promises greater autonomy by ‘tear[ing] down administrative, financial, philosophical and practical barriers’.^27^ Whilst this is a promising development for further developing new care models in those systems, there also needs to be consideration from national leaders about how to support areas at a more formative stage of development to develop the necessary capabilities to make these changes happen.

## Conclusions

The new care models programme in England was an attempt to do something different to make change happen, by providing support for local leaders to enable them to collaborate to improve care for their local populations. The 10 lessons we devised from the local vanguard leaders’ experiences emphasise the importance of developing relationships and building capability locally to enable areas to continuously develop and test new ideas. Proliferation of this approach to change could have a substantial impact on the health and care of the population and, in particular, on the lives of people who currently fall through the gaps created by service fragmentation.
